# Letter to the editor in response to Shih-Wei et al

**DOI:** 10.1016/j.tjpad.2025.100197

**Published:** 2025-05-18

**Authors:** Lorenzo Blandi, Paola Bertuccio, Carlo Signorelli, Helmut Brand, Timo Clemens, Cristina Renzi, Anna Odone

**Affiliations:** a School of Public Health, Vita-Salute San Raffaele University, Milan, Italy; b Department of International Health, CAPHRI Public Health and Primary Care Institute, Maastricht University, Maastricht, the Netherlands; c School of Public Health, Department of Public Health, Experimental and Forensic Medicine. University of Pavia, Pavia, Italy; d Medical Direction, Fondazione IRCCS Policlinico San Matteo, Pavia, Italy

Dear Editor,

We read with interest the comments made by Shih-Wei and colleagues to our article titled “Herpes zoster as risk factor for dementia: a matched cohort study over 20 years in a 10-million population in Italy” [1]. The authors raised as major point an important methodological challenge in studying the association between severe Herpes Zoster (HZ) and dementia, particularly regarding the influence of early dementia diagnoses within the first year after HZ hospitalization.

Recently, a 21-year population-based cohort study from Denmark reported similar patterns, trying to limit biases related to the first year of follow-up [2]. This study performed several sensitivity analyses, dividing follow-up in shorter and variable periods. The authors found that people with HZ have no association, or a slighted reduced incidence of dementia compared to people in the general population without HZ, reporting a hazard ratio of 0.98 (95% CI 0.92–1.04) in the first year of follow-up, and of 0.93 (95% CI 0.90–0.95) in the 1–21 years period. On the other hand, only within the first year of follow-up, they found a significant association among people exposed to HZ with the cranial nerve involvement. These results were consistent to another Taiwanese study, considering the Ophthalmic HZ [3].

By contrast, our 23-year population-based study followed a similar approach, improving some methodological aspects. First of all, we included only the most severe cases, seeking for acute care. Subsequently, as discussed in our manuscript, we considered the outcome occurrence both over the 0–1, 0–10, 1–10, 10–23 and 0–23 follow-up periods. This aimed to limit the biases occurring in the 0–1 period. Indeed, we calculated the 1–10 follow-up period (excluding the first year of follow-up, when more medical investigations could increase the likelihood of detection bias) and reported a still higher hazard ratio of 1.16 (95% CI 1.09–1.24) in comparison with the general population (group 1 vs. group 2). In addition, this higher risk remained significant, i.e. of 1.11 (95% CI 1.04–1.19), when comparing both people hospitalised with HZ and without HZ (group 1 vs. group 3). Indeed, as additional strategy, our study compared three different populations.

In our opinion, these different approaches provide alternative strategies and highlight the complexity of studying the temporal relationship between HZ and dementia incidence. Currently, several studies explored this relationship, reporting contradictory results. Researchers should read carefully all the different approaches and – in our opinion – focus on further aspects that are pivotal:

1. Data sources, looking for record linked data in integrated registries from different levels (e.g. primary care, hospital care, etc.) and sectors (e.g. social, health, environment) of our public health institutions. To this end, we have set up a regional scientific project called EPIDEM, from which we have already developed some studies using validated algorithms[4,5];
2. Methods, trying to refine models which can take into consideration the evolving social and health profiles (e.g. clinical comorbidities, social status, etc.) of individuals over long-time periods;
3. Preventive strategies and early interventions, considering the impact of vaccination and antiviral treatments in lowering the incidence of dementia among the adult population.

Once again, we thank the authors for their insightful comments on our study. Their observations will certainly contribute to advance the discussion and research in this area, and we remain committed to refining our understanding of the complex links between infectious diseases like HZ and the long-term risk of dementia.

## CRediT authorship contribution statement

**Lorenzo Blandi:** Conceptualization, Writing – original draft, Writing – review & editing. **Paola Bertuccio:** Conceptualization, Writing – review & editing. **Carlo Signorelli:** Conceptualization, Writing – review & editing. **Helmut Brand:** Conceptualization, Writing – review & editing. **Timo Clemens:** Conceptualization, Writing – review & editing. **Cristina Renzi:** Conceptualization, Writing – review & editing. **Anna Odone:** Conceptualization, Writing – original draft, Writing – review & editing.

## Declaration of competing interest

The authors declare the following financial interests/personal relationships which may be considered as potential competing interests: Lorenzo Blandi reports a relationship with Maastricht University that includes:. If there are other authors, they declare that they have no known competing financial interests or personal relationships that could have appeared to influence the work reported in this paper.
